# Lupin Peptides Modulate the Protein-Protein Interaction of PCSK9 with the Low Density Lipoprotein Receptor in HepG2 Cells

**DOI:** 10.1038/srep29931

**Published:** 2016-07-18

**Authors:** Carmen Lammi, Chiara Zanoni, Gilda Aiello, Anna Arnoldi, Giovanni Grazioso

**Affiliations:** 1 Department of Pharmaceutical Sciences, University of Milan, Milan, Italy

## Abstract

Proprotein convertase subtilisin/kexin type 9 (PCSK9) has been recently identified as a new useful target for hypercholesterolemia treatment. This work demonstrates that natural peptides, deriving from the hydrolysis of lupin protein and absorbable at intestinal level, are able to inhibit the protein-protein interaction between PCSK9 and the low density lipoprotein receptor (LDLR). In order to sort out the best potential inhibitors among these peptides, a refined *in silico* model of the PCSK9/LDLR interaction was developed. Docking, molecular dynamics (MD) simulations and peptide binding energy estimations, by MM-GBSA approach, permitted to select the two best candidates among tested peptides that were synthesized and evaluated for their inhibitory activity. The most active was **P5** that induced a concentration dependent inhibition of the PCSK9-LDLR binding, with an IC_50_ value equal to 1.6 ± 0.33 μM. Tested at a 10 μM concentration, this peptide increased by 66 ± 21.4% the ability of HepG2 cells to take up LDL from the extracellular environment.

An increased level of low-density lipoproteins (LDL) predisposes to the development of cardiovascular disease (CVD) and stroke. Currently, the main agents for lowering LDL levels are statins. They reduce the regulatory pool of intracellular cholesterol by competitively inhibiting HMGCoAR, the rate-limiting enzyme of endogenous cholesterol biosynthesis. This in turn activates the LDL receptor (LDLR) transcription, a process under the control of sterol regulatory element binding protein 2 (SREBP-2)^1^,^2^. The statin cholesterol-lowering action has been consistently shown to translate into fewer cardiovascular events^3^,^4^,^5^. Nevertheless, residual risk persists in a large portion of statin-treated individuals, owing to either inability to achieve desirable LDL levels^6^,^7^ or presence of other traits predisposing to CVD^8^,^9^. Moreover, intolerance to statins produces numerous side effects, such as myopathy with a spectrum of consequences ranging from myalgia to rhabdomyolysis^10^. For the treatment of these individuals, it is thus advisable to use alternative or adjunctive therapies. In this context, proprotein convertase subtilisin/kexin type 9 (PCSK9) has been recently identified as a new useful target for hypercholesterolemia treatment^11^.

The human 22-kb gene *PCSK9* is located on the small arm of chromosome 1p32 and contains 12 exons and 11 introns^11^. The gene encodes a 692-residue extracellular protein named PCSK9 that is expressed primarily in liver, kidney, and intestine^12^ and plays an important role in regulating the degradation of hepatic LDLR^13^,^14^. Notably, since PCSK9 and LDLR are co-regulated by SREBP-2^1^, increased PCSK9 expression in response to statin-induced cellular cholesterol depletion may limit the efficacy of statin treatment^15^,^16^. The development of therapies that inhibit PCSK9 function holds promise for improved management of hypercholesterolemia and CVD risk. In particular, evidence supports the direct binding of secreted PCSK9 to the LDLR, resulting in the receptor degradation^17^,^18^. The PCSK9 binding site in the LDLR is located at the first epidermal growth factor-like repeat (EGF-A) of the extracellular domain^13^ and this binding is required for LDLR degradation.

In light of these observations and the fact that circulating PCSK9 may cause the degradation of hepatic LDLR, this protein is considered an attractive target for LDL-cholesterol lowering drugs. The current inhibitors under development are based on different strategies, i.e. monoclonal antibodies (mAbs) (such as evolocumab and alirocumab)^19^, gene silencing compounds^20^, and natural product such as berberine^21^, and peptidomimetics^22^,^23^. The most investigated strategy advancing to clinical development is the blockade of PCSK9 interaction with LDLR *via* mAbs. In fact, PCSK9 is an ideal antibody target, since it circulates in the blood and has only modest binding affinity to cell surface LDLR. Additionally, its main interaction site is an exposed ∼500-Å^2^ slightly convex region readily accessible for antibody binding.

The ability of PCSK9 to mediate LDLR degradation involves protein-protein interactions (PPIs) between LDLR and the PCSK9 prodomain^24^, catalytic domain^25^, and C-terminal Cys-rich domain (CRD)^12^,^26^,^27^,^28^. In this context, PPIs, which are inherently challenging small molecule targets, have been successfully inhibited by peptides, which can recapitulate key protein contacts^29^.

Lupin protein consumption provides some health benefits in the area of hypercholesterolemia prevention^30^. Studies in animal models^31^,^32^ and in the clinics^33^,^34^ have shown that the protein itself is a main hypocholesterolemic agent. In this scenario, considering that proteins are hydrolyzed in the gastrointestinal apparatus, a recent paper^35^ has demonstrated that tryptic and peptic peptides deriving from lupin protein hydrolysis are able to modulate cholesterol metabolism in HepG2 cells by inhibiting HMGCoAR via a statin-like mechanism. Another investigation in human Caco-2 cells has provided evidence that at least some of these peptic and tryptic peptides may be absorbed in the small intestine, since they are transferred from the apical (AP) to the basolateral (BL) compartment in differentiated Caco-2 cells grown in a two-compartment system^36^. Table 1 shows the sequences of the transferred peptides that here will be referred to as “absorbable lupin peptides”. Finally, another study in mild hypercholesterolemic subjects has demonstrated that the level of circulating PCSK9 is reduced in a statistically significant way after a 4-week consumption of a lupin protein isolate (30 g protein/day)^37^. The same paper reported also an investigation in human HepG2 cells showing that lupin peptides reduce the synthesis and secretion of mature PCSK9 as well as the level of hepatic nuclear factor 1-alpha (HNF1-alpha)^37^.

In this context, the present work was aimed at evaluating whether “absorbable lupin peptides” may directly inhibit the PCSK9-LDLR PPI. A second objective was to sort out the best potential inhibitors among “absorbable peptides” by a computational protocol specifically developed for this goal. Docking, molecular dynamics (MD) simulations and peptide binding energy estimations, by MM-GBSA approach, permitted to select the best candidates among peptic and tryptic peptides that were synthesized and evaluated for their inhibitory activity of the PCSK9-LDLR interaction and subsequent capability of reducing the LDL uptake by HepG2 cells.

## Results

### Absorbed peptic and tryptic lupin peptides maintain their capacity to interfere with the PCSK9/LDLR PPI

As already explained in the introduction, peptic peptides (**P-tot**) and tryptic peptides (**T-tot**) from lupin protein reduce the synthesis and secretion of mature PCSK9 in HepG2 cells^37^. In parallel, other experiments, performed in Caco-2 cells grown in two-compartment systems, have indicated that some of these peptic and tryptic peptides might be absorbed in the small intestine^36^. Precisely, Caco-2 cells have been incubated for 4 h in the AP chamber with 500 μl of a 1.0 μg/μl solution of **P-tot** or **T-tot** peptides and, at the end of treatment, BL solutions (700 μl) have been collected and absorbed peptic and tryptic peptides identified by HPLC-Chip ESI-MS/MS^37^. The BL solutions were then dried and diluted to 100 μl to obtain the solutions **P-abs** and **T-abs** at the concentration of 0.25 and 0.32 μg/μl, respectively. Both solutions were used for the present experiments.

**Figure 1:**
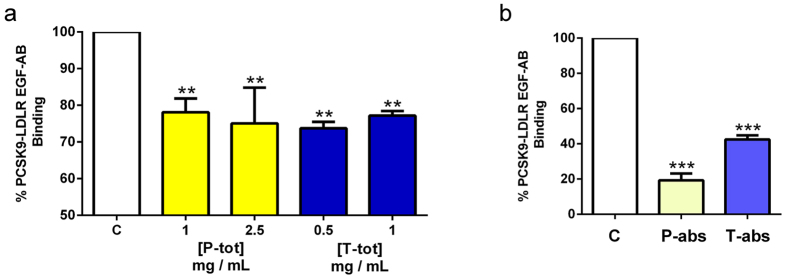
Effects of peptic and tryptic lupin peptides on the PCSK9-LDLR binding inhibition. (**a**) *In vitro* screening of the inhibitory activity of total lupin peptides, i.e. **P-tot** (1.0 and 2.5 μg/μl) and **T-tot** (0.5 and 1.0 μg/μl), on PCSK9-LDLR PPI; and (**b**) of absorbed lupin peptides, i.e. **P-abs** and **T-abs** (diluted 1:20) (**b**). The commercial assay provides a plate pre-coated with a recombinant LDLR-AB domain, which is the PCSK9 binding site. For screening direct inhibitors of the PCSK9-LDLR interaction, lupin peptides were added to the proper amount of His-tagged PCSK9 in each well coated with LDLR-AB domain. Biotinylated anti-His-tag monoclonal antibody specifically reacted with recombinant His-tagged PCSK9 trapped with LDLR-AB domain immobilized on the microplate surface. The inhibitory effects were analyzed measuring the amount of His-tagged PCSK9 on the well. In particular, the inhibitory effect, calculated as % PCSK9-LDLR binding inhibition, determines a reduction of absorbance measured at 450 nm using the Synergy H1 fluorescent plate reader from Biotek *versus* vehicle control. Data points represent averages ± SEM of three independent experiments in duplicate. ***p < 0.0001 **p < 0.001 versus C. C, control vehicle.

The first experiment had the objective of comparing the ability of total and absorbed peptides to inhibit the PCSK9-LDLR binding. *In vitro* experiments were performed using a solid-phase binding assay between recombinant His-tagged PCSK9 and recombinant LDLR-AB domain. The results are shown in Fig. 1. **P-tot** peptides reduced the PCSK9-LDLR binding by 22 ± 6.5% and 25 ± 13.7% (*p* < *0.001*) at 1.0 and 2.5 μg/μl, respectively (**Chart 1A**), **T-tot** peptides by 26 ± 3% and 23 ± 2% at 0.5 and 1.0 μg/μl, respectively, whereas **P-abs** peptides (12.5 ng/μl) and **T-abs** peptides (16.0 ng/μl) inhibited the PCSK9-LDLR interaction by 81 ± 7.6% and 58 ± 4.1% (*p* < *0.0001*), respectively, *versus* the untreated sample (**Chart 1B**).

### In silico model of the human PCSK9

**Figure 2:**
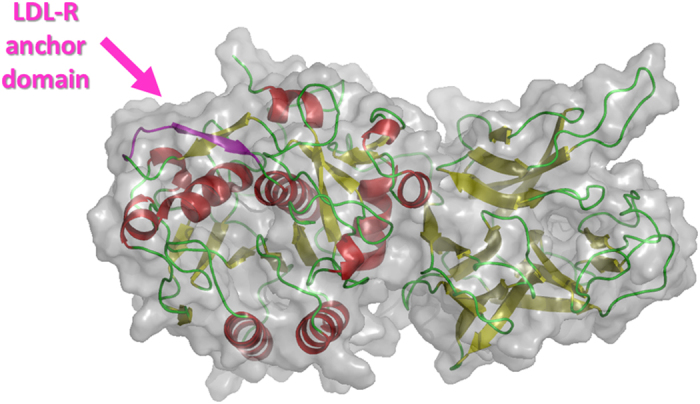
Representation of the PCSK9 model used for simulations. Water molecules have been omitted for sake of clarity.

The 3D structure of PCSK9 was modeled and refined following the procedure described in the “Methods” section. The PCSK9 model (Figs 2 and S1 in the Supporting Information) was selected on the basis of the internal Modeller9.15^38^ and DOPE scoring function results. It was further validated applying the QMEAN6^39^ algorithm acquiring a score of 0.7. For this scoring function, quality factors may fall in the range between 0 and 1, with higher values for better models. However, low percentages of residues were in disallowed (0.7%) and generously allowed regions (0.7%) of the Ramachandran plot. Nevertheless, residues, whose phi-psi angle values were outside the allowed regions of the plot, belonged to peripheral loops and were further equilibrated by means of MD simulations.

At the end of simulations, the thermal stability of the PCSK9 model was ascertained by analyzing the atomic fluctuations per residue plotted *versus* the residue number during the MD simulations. Remarkably, the model displayed a good geometrical stability showing a mean RMSF of Cα atoms of 1.21Å (Figure S2). Moreover, residues displaying the lowest thermal stability of the backbone atoms were those not well-solved by X-ray studies, or belonging to loop connecting secondary structures.

In light of these results, this PCSK9 model was chosen as target protein for docking and further employed for lupin peptides binding energy calculation, by a MM-GBSA approach.

### The lupin protein models

**Figure 3:**
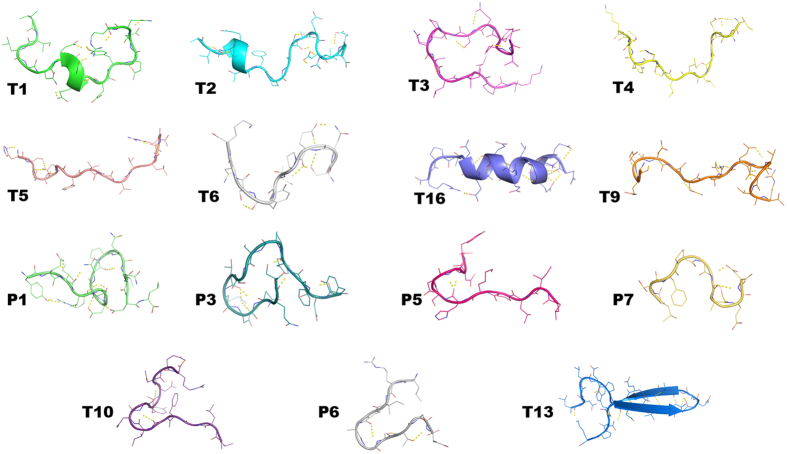
Three-dimensional structure of lupin small-peptides at the end of MD simulations and subsequent energy minimization.

Investigated lupin peptides belong to the lupin proteins β1- and β2-conglutins, α-conglutin and δ-conglutin. Their computational models were created by homology modeling techniques following the procedure described in the “Methods” section. The high percentange of sequence homology found with the employed templates permitted to achieve models with reasonable quality, additionally supported by MD simulations. At the end of simulations, the energy minimized structures of small peptides **T1**, **T2**, and **T16** showed helix conformation, **T3**, **T4**, **T5**, **T6**, and **T9** were folded as ramdom coils, **P1**, **P3**, **P5**, **P7**, **T10**, and **P6** showed γ-turn conformations, while peptide **T13** was shaped as β-hairpin (Fig. 3).

In order to establish which peptides were mainly responsible for the PCSK9/LDLR PPI inhibition, these models were used for docking calculations into the LDLR anchor domain of PCSK9. The subsequent MD simulations and final MM-GBSA calculations permitted estimating their theoretical binding energy values on PCSK9 reported in Table 2. The lowest theoretical binding energy values were calculated for **T9** and **P5**, i.e. a tryptic and peptic peptide.

**Figure 4:**
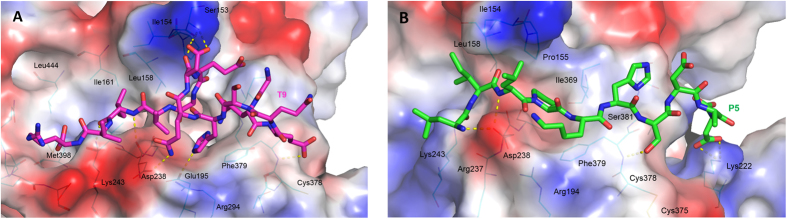
Docking and MD simulations results for **T9** (panel A) and **P5** (panel B). **T9** and **P5** are represented as magenta and green stick models, respectively. The showed conformations of **T9** and **P5** were retrieved minimizing MD frames in which the systems displayed geometrical stability. PCSK9 is displayed as cyan thin stick model and only residues within a radius of 4 Å from docked peptides are shown and labeled in the pictures. PCSK9 surface is colored according to the atoms electrostatic charges (blue for positive and red for negative), suitably calculated by Pymol tools.

**Figure 5:**
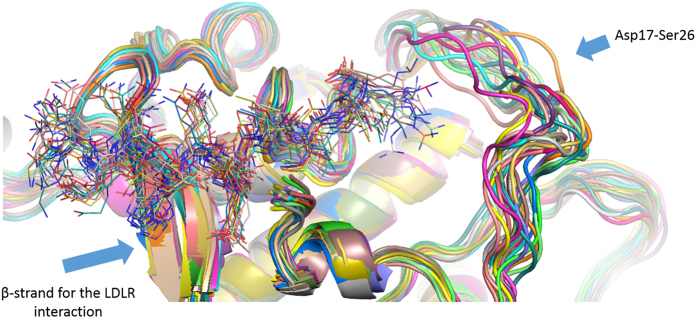
Representation of PCSK9 trajectory frames acquired during MD simulations and selected for MM-GBSA analysis. Different conformations of **T9** are represented as thin stick models while PCSK9 is represented as cartoon. Arrows indicates the most flexible portion of the enzyme (From Asp17 to Ser26) and the localization of the site where the EGF-A domain of LDLR can be anchored in physiological conditions.

For a deep exploration of the potential interactions these peptides create with the biological counterpart, we visually inspected by Pymol the PCSK9/**T9** complex resulted minimizing a frame of MD simulations in which the system showed geometrical stability. This analysis revealed the presence of several polar and van der Waals interactions between the peptide and the enzyme counterparts. In particular, **T9** is able to create hydrogen bonds by Glu3, His6, Gln7, Asp8 and Ile12 with the PCSK9 residues Cys378, Glu195, Asp238, Ser153 and Lys243. Moreover, the side chain of Ile12 in **T9** is projected in a hydrophobic pocket shaped by Ile161 and Leu158 (Fig. 4A). Interestingly, MD simulations suggested that C-terminal Arg14 is immersed in a cavity with variable size due to the high conformational mobility of the protein loop formed by sequence from Asp17 to Ser26 (Fig. 5). This conformational mobility explains why the same protein portion was not solved by X-ray studies. However, by way of our developed model, this area could represent the target for designing new potent ligands or peptides.

On the other hand, **P5** produces a hydrogen bond assisted salt bridge by means of C-terminal Asp11 with Lys222 of the enzyme, while its Leu1, Leu3, and Ser7 can produce a supplementary hydrogen bond network with Lys243, Asp238, and Phe379 of PSCK9, respectively (Fig. 4B).

### Isolated lupin peptides inhibit the PCSK9-LDLR binding

**Figure 6:**
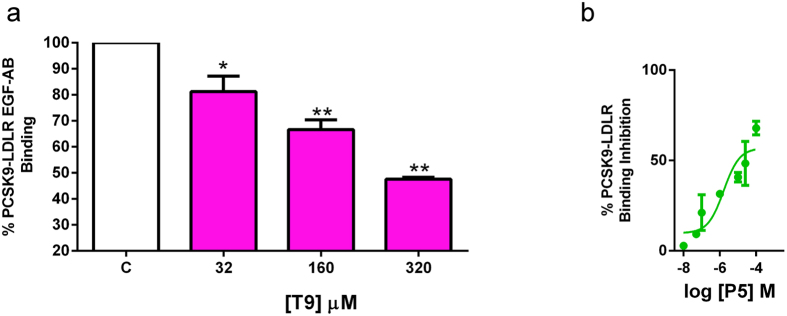
Peptides T9 and P5 inhibit the PCSK9-LDLR binding. Inhibitory effects of (**a**) **T9** (32, 160, and 320 μM) and (**b**) **P5** (0.001–100 μM) on the PCSK9-LDLR PPI *in vitro*. The employed assay provides a plate pre-coated with a recombinant LDLR-AB domain, which is the PCSK9 binding site. For screening, the peptides were added to proper amount of His-tagged PCSK9 in each well coated with LDLR-AB domain. Biotinylated anti-His-tag monoclonal antibody specifically reacts with recombinant His-tagged PCSK9 trapped with LDLR-AB domain immobilized on the microplate surface. The inhibitory effects of **T9** and **P5** were analyzed measuring the amount of His-tagged PCSK9 on the well. The **T9** and **P5** inhibition, calculated as % PCSK9-LDLR binding inhibition, was measured from the absorbance reduction at 450 nm, using the Synergy H1 fluorescent plate reader (Biotek) *versus* vehicle (control). **P5** showed a concentration-response behavior, with an IC_50_ equal to 1.6 μM. Data points represent averages ± SEM of three independent experiments in duplicate. *p < 0.05 **P < 0.001 versus C. C, vehicle.

In order to assess the efficacy of the bioinformatics prediction, **T9** and **P5**, the best potential inhibitors among peptic and tryptic peptides identified through computational studies, were synthesized and tested for their capacity to inhibit the PCSK9-LDLR binding. **T9** significantly reduced the PCSK9-LDLR binding by 19 ± 8.47%, 33 ± 5.33%, and 53 ± 1.02% (*p* < *0.05* and *p* < *0.001*) at 32, 160, and 320 μM concentrations, respectively, *versus* the untreated sample (Fig. 6a). **P5 (**tested in the range 0.001–100 μM) induced a concentration dependent inhibition of the PCSK9-LDLR binding, with an IC_50_ value equal to 1.6 ± 0.33 μM (Fig. 6b).

### Through the inhibition of PCSK9-LDR PPI, T9 and P5 increase the ability of HepG2 cells to uptake LDL from the extracellular environment

**Figure 7:**
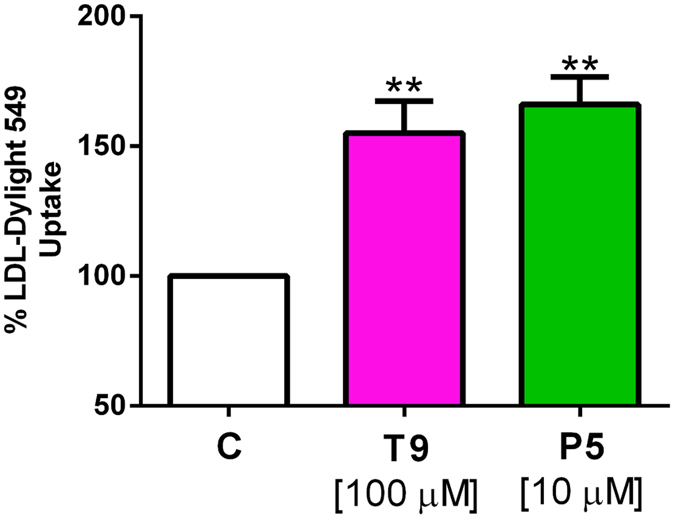
Fluorescent LDL-uptake assay after lupin peptide treatments of HepG2. Cells (3 × 10^4^) were treated with **T9** (100 μM) and **P5** (10 μM) for 24 h. LDL-Dylight 549 (10 μg/ml) was incubated for an additional 2 h. Excess LDL-Dylight 549 was removed and cells were washed 2 times with PBS. Specific fluorescent LDL-uptake signal was analyzed by Synergy H1 (Biotek). Data points represent averages ± SEM of three independent experiments in triplicate. **p< 0.001 *versus* C. C, vehicle.

For a deeper insight of the activity of **T9** and **P5**, the change of the functional capability of HepG2 cells to uptake extracellular LDL was investigated after treatment with these peptides by performing fluorescent-LDL uptake experiments. As shown in Fig. 7, each peptide increased the LDL-uptake *versus* the control in a statistically significant way (*p* < *0.001*). In fact, the treatment with **P5** (10 μM) led to an increase of the LDL-uptake by 66 ± 21.4%, whereas that with **T9** (100 μM) increased the LDL-uptake by 55 ± 24.4%, *versus* the untreated sample. Therefore, both **P5** and **T9** are able to improve the HepG2 ability to absorb extracellular LDL, although their potency is very different, being the latter about ten times less active than the former.

Recently, we have demonstrated that HepG2 cells secrete 141 ng/ml of mature PCSK9 in physiological conditions^37^. These results indicate that the capacity of **T9** and **P5** to impair the binding of secreted PCSK9 to the LDLR stabilizes the active LDLR on cell membrane, leading to an improved ability of hepatic cells to uptake extracellular LDL with a final hypocholesterolemic effect.

### Quantification of P5 in the sample P-abs

The excellent activity of **P5** suggested its quantification in the **P-abs** sample. This was obtained by HPLC-MS/MS by building the calibration curve injecting the synthetic standard: the **P5** concentration in the **P-abs** solution was calculated to be 28.64 ± 0.43 ng/μl, corresponding to 11.5% of the total peptic peptides absorbed by CaCo-2 cells.

## Discussion

The discovery of PCSK9 in 2003^40^ opened a new scenario in the lipid field. In fact, whereas previously statins were considered the best hypocholesterolemic drugs, subsequently PCSK9 inhibitors became another therapeutic option. In this context, numerous studies have focused their attention on mAbs^19^, gene silencing compounds^20^, peptidomimetics^22^, and berberine^21^. At the best of our knowledge, instead, this is the first paper providing experimental evidence that some peptides deriving from food protein hydrolysis may function as effective inhibitors of the PCSK9-LDLR PPI.

Our investigation was focused on lupin protein, since a very recent paper has shown that in hypercholesterolemic subjects, who had consumed dietary bars containing lupin protein for a month, the total cholesterol decrease was accompanied by a parallel decrement of circulating PCSK9^37^. Other relevant evidences available before starting the present investigation were the following: 1) peptic and tryptic peptides, obtained digesting a total protein extract from lupin seed with pepsin and trypsin are able to inhibit the interaction of PCSK9 with the LDLR in an *in vitro* system^37^; 2) some of these peptides, identified by HPLC-ESI-MS/MS, are absorbed in a Caco-2 model of the small intestine^36^.

These evidences prompted us to investigate whether the absorbed peptides (**P-abs** and **T-abs**) maintained the inhibitory activity of **P-tot** and **T-tot**, i.e. whether at least some bioactive peptides had been absorbed. In this context, the demonstration that the capacity of these peptides to inhibit the PCSK9/LDLR interaction is not impaired by the absorption is certainly a main achievement of this work. The fact that at least some bioactive peptides are absorbed is not obvious, considering that only part of the original peptides were absorbed in the Caco-2 model^36^, since at the end of treatment some were still detectable only in the AP compartment and other disappeared in both compartments owing to metabolic degradation.

A natural development of this research was the application of *in silico* tools for potentially sorting out the most bioactive peptides. The first step was the modeling a refined human PCSK9 3D-structure, starting from X-ray available literature data^23^. Afterward, the peptides 3D-structures were calculated and refined starting from the structures of the proteins to whom they belong. Their conformations are very variable, since three have helix conformations, five γ-coil conformations, one β-hairpin conformation, and six are random coils. Finally, the docking study permitted to estimate their theoretical binding energy values on PCSK9 and therefore to get a classification in which **T9** was the best candidate among tryptic peptides and **P5** the best among peptic peptides. Interestingly, both derive from the hydrolysis of lupin β-conglutin, a 7S vicilin-like storage globulin. **T9** consists of 14 amino acidic residues, whereas **P5** of 10 amino acidic residues.

The following experiments, performed on the synthetic peptides, confirmed that indeed they are able to impair the interaction of PCSK9 with the LDLR. **P5** resulted to be the most active, with an IC_50_ value equal to 1.6 μM, whereas **T9** was much less active, since its IC_50_ falls around 320 μM. The experiment on the ability of HepG2 cells to uptake LDL from the extracellular environment confirmed the better efficacy of **P5**. This is not completely in agreement with the MM-GBSA calculations, which predicted a higher affinity for **T9** than **P5**. This inaccuracy might be due to the omitted estimation of the entropic contribution to the binding free energy of peptides. In practice, the entropic contribution to the binding free energy could be higher than that of **T9**, explaining the discrepancy between the calculation and the experimental results. Unfortunately, computational techniques do not permit to get an accurate prediction of the entropic contribution value yet. Available tools are computationally very demanding, requiring highly energy minimized structures for “normal mode” analysis, or large amount of conformations for “quasi-harmonic” analysis^41^,^42^,^43^. In any case, our calculations permitted to sort out at least one lupin peptide with a great potential interest as inhibitor of the interaction of PCSK9 with the LDLR, owing to the very low IC_50_ value equal to 1.6 μM.

It is useful to observe that Pep2-8 (Ac-TVFTSWEEYLDWV-amide), the best inhibitor singled out by Zhang and coworkers in a recent paper^23^, has an IC_50_ value equal to 0.81 ± 0.08 μM, i.e. only slightly better than that of **P5**. Interestingly, Pep2-8 contains 13 amino acid residues and its C-terminal truncated analogues loose activity, whereas **P5** contains only 10 residues. **P5** is thus one of the shortest peptides ever described in literature endowed with this specific activity.

**Figure 8:**
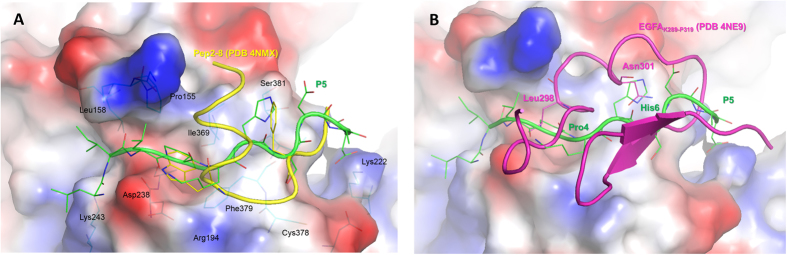
(**A**) Superimposition of PCSK9/**P5** and PCSK9/Pep2-8 (PDB code 4NMX) complexes. **P5** is represented as green stick and cartoon models, whereas Pep2-8 is colored in yellow. (**B**) Superimposition of PCSK9/**P5** and PCSK9/EGF-A (residues from Ly289 to Pro319, PDB code 4NE9) complexes. **P5** remains represented as green stick and cartoon models, whereas EGF-A domain of LDLR is colored magenta. Both panels show the PCSK9 surface colored according to the atoms electrostatic charges (blue for positive and red for negative), suitably calculated by Pymol tools.

The superimposition of PCSK9/**P5** complex, resulted by minimizing a frame of MD simulations in which the system showed geometrical stability, with the X-ray structure of PCSK9/Pep2-8 (PDB code 4NMX) revealed that the α-helix of Pep2-8 was found in proximity of the PCSK9 N-terminal domain (Ser153). The Leu-Ile-Leu sequence of **P5** was able to occupy a basin shaped by Ile154, Leu158 and Lys243 (Fig. 8A) while the side chain of **P5** Pro4 was perfectly aligned on the one belonging to Pep2-8 Trp6, and both residues occupied the pocket sized by PCSK9 Phe379, Ala239, and Asp238. Additionally, the side chain of **P5** His6 was located into the PCSK9 area close to Ile369, as the side chain of Pep2-8 Phe3. This observations may open the way to the design of new and more potent derivatives (small molecules or peptides), able to occupy the Ile154, Leu158 and Lys243 solvent-exposed pocket. As well, **P5** sequence could be optimized by performing a virtual alanine-scanning aiming to predict which residues efficiently contribute to the binding of the peptide on PCSK9. Then, residues could be suitably mutated into new terms showing higher complementarity with the PCSK9 active site.

Remarkably, also the superimposition of **P5** on the EGF-A domain of LDLR co-crystallized with PCSK9 (PDB code 4NE9) showed a good overlapping. In fact, Pro3 and His6 of **P5** were able to mimic very well the interactions played by the side chains of EGF-A Leu298 and Asn301 on PCSK9. Additionally, the side chain of **P5** Ser7 was in line with the oxygen atom of EGF-A Cys308, creating a hydrogen bond with the backbone of PCSK9 Phe379. Taking into account these considerations, it is possible to affirm that **P5** is able to mimic some key interactions played by Pep2-8 and EGF-A on PCSK9. This may explain the efficient biological activity displayed by **P5**.

Nevertheless, the real conformations of the PCSK9/small peptides complexes could be different from those here described. The reasons of this uncertainty are due to the use of molecular mechanics methods, the limited sampling of MD simulations, and the creation of homology models of the lupin proteins. However, we have applied well-validated molecular modeling procedures in order to decrease as possible the degree of uncertainty.

Finally, it is important to recapitulate the very original approach of our investigation:

1. detection of a PCSK9 decrease in the plasma of hypercholesterolemic subjects at the end of a dietary intervention study based on dietary bars containing lupin protein;
2. demonstration that lupin peptides reduce the PCSK9 synthesis and secretion in HepG2 cells;
3. identification of lupin peptides which are absorbed in Caco-2 cells;
4. demonstration that they retain the original capacity of impairing the PCSK9/LDLR interaction;
5. classification of their potential activity by docking studies with the PCSK9-LDLR complex and selection of the best candidates;
6. identification of the very potent peptide **P5**.

Owing to the unusual process of the identification and its original sequence, indeed **P5** may be a promising candidate for designing new potent inhibitors of the PCSK9/LDLR PPI. In conclusion, this is the first experimental evidence that a peptide deriving from a plant protein has this specific biological activity.

## Methods

### Preparation of the pepsin and trypsin peptide mixtures

The peptic (**P-tot**) and tryptic (**T-tot**) hydrolyzates from lupin protein are the same used in a previous paper, in which their capability to modulate cholesterol metabolism in HepG2 cells was investigated^35^. The procedure for protein extraction from *Lupinus albus* (cultivar Ares) seeds, the conditions of enzymatic hydrolysis, and the final composition of the resulting peptide mixtures are reported in detail in the same paper. In the tryptic sample, 12 peptides were assigned to *L. albus* vicilin-like protein (Q53HY0), 10 peptides to *L. albus* beta‐conglutin precursor (Q6EBC1), 4 peptides to *Lupinus angustifolius* conglutin beta (B0YJF8)], 4 peptides to *L. angustifolius* conglutin-beta (Q53I55)], and 2 to *L. albus* conglutin-delta seed storage protein precursor (Q99235). In the peptic hydrolysate, 21 peptides were assigned to *L. albus* vicilin-like protein (Q53HY0), 18 peptides to *L. albus* beta‐conglutin (Q6EBC1), 7 peptides to *L. angustifolius* conglutin-alpha 3 (F5B8V8], and 8 peptides to *L. albus* conglutin-gamma (Q9FSH9). These mixtures were submitted to absorption experiments in Caco-2 cells grown in two-compartment trans-well systems^36^. The absorbed peptic (**P-abs**) and tryptic peptides (**T-abs**), i.e. those transferred in the basolateral compartments, are shown in Table 1. The details for the preparation and quantification of the peptides and the analytical procedure for their identification based on mass spectrometry are described in detail in that paper^36^.

### *In vitro* PCSK9-LDLR binding assay

**P-tot** peptides (1.0 and 2.5 μg/μl), **T-tot** peptides (0.5 and 1.0 μg/μl), **P-abs** (12.5 ng/μl) and **T-abs** peptides (16.0 ng/μl) were tested using the *in vitro* PCSK9-LDLR binding assay (CycLex Co., Nagano, Japan) following the manufacture instructions. Briefly, plates are pre-coated with a recombinant LDLR-AB domain, which contains the binding site for PCSK9. Before starting the assay, tested peptides and/or the vehicle were diluted in reaction buffer and added in microcentrifuge tubes. Afterwards, the reaction mixtures were added in each well of the microplate and the reaction was started by adding His-tagged PCSK9 wild type solution (3 μl). The microplate was allowed to incubate for 2 h at room temperature (RT) shaking at 300 rpm on an orbital microplate shaker. Subsequently, wells were washed 4 times with wash buffer. After the last wash, the biotinylated anti-His-tag monoclonal antibody (100 μl) was added and incubated at RT for 1 h shaking at 300 rpm. After incubation, wells were washed for 4 times with wash buffer. After the last wash, 100 μl of HRP-conjugated streptavidin were added and the plate was incubated for 20 min at RT. After incubation, wells were washed 4 times with wash buffer. Finally, the substrate reagent (tetra-methylbenzidine) was added and the plate was incubated for 10 min at RT shaking at ca. 300 rpm. The reaction was stopped with 2.0 N sulfuric acid and the absorbance at 450 nm was measured using the Synergy H1 fluorescent plate reader (Biotek, Bad Friedrichshall, Germany).

### The human PCSK9 model

Molecular modeling studies started retrieving the PCSK9/LDLR complex (PDB code 4NE9, resolution 2.6 Å) from Protein Data Bank (PDB)^44^. The unsolved regions of PCSK9 were modeled by Modeller9.15^38^, taking into account also the crystal structure stored with PDB code 3P5C^45^. This structure has reduced amount of unsolved regions but lower resolution (4.2 Å). One hundred models were created: the most accurate one was chosen considering the results of the DOPE scoring function and the quality factors acquired by means of Procheck^46^ and QMEAN6 score, provided by SWISSMODEL server/Protein Structure & Model Assessment Tools^39^.

Furthermore, in order to better refine the rough PCSK9 model, energy minimization and MD simulations were performed. The model was solvated with almost 25,000 water molecules (Amber12 package)^47^, creating a system composed by more than 79,000 atoms. FF03^48^ version of the AMBER force field was used for the protein, while the TIP3P model^49^ was used to explicitly represent water molecules. Van der Waals and short-range electrostatic interactions were estimated within 12 Å cutoff, while the long-range electrostatic interactions were included by using the particle mesh Ewald method^50^.

Bonds involving hydrogen atoms were constrained using the SHAKE algorithm^51^, enabling the use of a 2 fs time step. After a preliminary minimization run, where only the solvent was optimized, the model was geometrically optimized starting from the side chain conformations and then continuing with the whole protein. Prior to starting the MD simulations, the system was equilibrated for 40 ps at 300 K in isocore conditions (NVT), then isothermal-isobaric ensemble were carried out at 300 K. Berendsen’s coupling algorithm^52^ was active to maintain the pressure (1 atm) for both solvent and solute molecule, while the coupling constant was set to 1.5 ps. Periodic boundary conditions were applied in the MD simulations and, in the production run, 100 ns of MD simulations were performed by *pmemd.cuda* module of AMBER12. Then, the acquired MD trajectory was examined by visual inspection with VMD^53^, ensuring that the thermalization did not cause any structural distortion.

### Models of small peptides

The lupin globulins β1- and β2-conglutins are the major sources of small peptides considered in this study, i.e. **T1–T6, T9–T10, P1**, **P3**, **P5**, **P6-P7** (Table 1). In order to build their computational models, it was initially decided to create the 3D structure of complete β1- and β2-conglutins. Then, the portions matching the sequence of the target small-peptides were extracted and singularly geometrically refined by MD simulations in a water environment.

Unfortunately, structural information of both β-conglutins are not available in Protein Data Bank, thus homology-modeling techniques were applied to build these models. In particular, the SWISSMODEL server^54^ found the optimal templates for the target sequences and Modeller9.15^38^ was used to build the final 3D models. Two templates, soybean β-conglycinin (PDB code 1UIK, 56.4% identity, 85.6% similarity)^55^ and AraH1, a vicilin-like protein from peanut (PDB code 3S7E^56^, 52.9% identity and 83.4% similarity, Figures S3 and S4 in Supporting materials) were employed for the construction of the β2-conglutin models. Whereas, the β1-conglutin model was built by homology modeling using the previously developed β2-conglutin model, due to their high sequence homology (94.3% sequence identity and 97.9% similarity).

**T13,** derived from α-conglutin, was built by the similar approach using as template soybean proglycinin (59.1% sequence identity and 83.0% similarity, Figure S5 in Supporting materials), for which three crystal structures (PDB codes 1UCX^57^, 1FXZ^58^ and 3KSC^59^) were available.

Finally, **T16**, deriving from δ-conglutin, was built using peanuts AraH2 protein (PDB code 3OB4) as template (50.0% sequence identity and 87.5% similarity, Figure S6 in Supporting materials).

All sequence alignments were performed by *BLOSUM 250* matrix and LALIGN server^60^, then Modeller9.15^38^ built one hundred models of each target protein. The best models were chosen accordingly with their quality parameters (DOPE scoring function and PROCHECK^46^).

Each model was then solvated, computationally parameterized as previously described for PCSK9, energy minimized, and submitted to MD simulations (20 ns), in order to geometrically equilibrate the systems. Finally, portions matching with each peptide were extracted and the suitable models were optimized again in water environment by additional 20 ns of MD simulations.

### Docking, MD simulations and peptide binding energy estimations

In order to establish which lupin peptides were the main responsible for the biological activity on PCSK9, their theoretical binding energies needed to be calculated. To this aim, docking studies, MD simulations and Molecular Mechanics-Generalized Born Surface Area (MM-GBSA) calculations were performed^41^. Docking of proteins is still challenging, due to their high flexibility. Several algorithms, also available by web-server, have been developed to this aim. For example, pepATTRACT^61^ and CABS-dock server^62^, based on a coarse-grained protein models, have been designed to efficiently take into account the protein conformational changes, obtaining satisfactory results. Additionally, FlexPepDock^63^,^64^ or HADDOCK^65^ are tools capable to refine the docking poses produced by different algorithms, in the respect of sets of experimental data. Nevertheless, also small molecules docking programs are valid alternative to these algorithms^66^. Here, we performed all-atoms docking calculations by GOLD5.2 algorithm^67^, taking into account all degrees of freedom of small peptides. Further MD simulations permitted both the optimization of the small peptide geometries into the PCSK9 EGF-A binding domain, and the acquiring of the MD trajectories useful for the binding free energy estimation, by MM-GBSA approach. In the docking calculations, fifty poses were generated for each peptide in the binding site recognized by the presence of the EGF-A domain of LDLR in the PCSK9 crystal structure (12 Å around PCSK9 Phe379). The conformations of the lupin small peptides resulted minimizing the last frame of MD simulations were used for docking. Peptides were docked considering all the degrees of freedom for the side chains and backbone torsion angles. At variance, peptide bonds were retained in “s-trans” conformations and the presence of internal H-bonds were detected and conserved during the docking calculations. PCSK9 were considered frozen and at the end of calculations, all generated poses were clustered by the complete-linkage method^68^. This is an agglomerative hierarchical clustering algorithm in which, initially, each element is located in a different singleton cluster. Clusters are then combined into larger ones until all elements are in the same cluster.

The clusters of solutions displaying RMSD values within 4 Å were selected and ranked depending on the ChemPLP^69^ score acquired by the representative solution, the one with the highest score in each cluster. Due to the very high degrees of freedom of each peptide, numerous clusters were created, showing similar scores but different binding modes. For this reason, the first three clusters of each peptides were visually inspected by Pymol and superimposed on the available X-ray structures of PCSK9 with EGF-A and Pep2-8 peptides. Then, the docking solutions of lupin peptides able to mimic their structural features were selected for further studies.

Accordingly, the obtained PCSK9/small peptide complexes were submitted to MD simulations, adopting the computational parameters previously described for PCSK9 model. Here, in the production runs, 50 ns of MD simulations for each system were recorded, permitting to acquire the trajectories useful for the subsequent binding energy calculations by the “single trajectory” protocol of the MM-GBSA approach^70^. The main advantage of this computational protocol is the use of an ensemble of structures (snapshots) accounting for more than one possible conformation of the protein/peptide complex. Therefore, the MM-GBSA methodology was not sensitive to the atomic details of single representative conformations obtained by docking methods. Furthermore, through the application of the conformational sampling to generate thermodynamic averages, one can also better simulate the protein-protein, or the protein-ligand, reciprocal adaptation, a phenomenon commonly referred to as the induced-fit^71^.

In this case, snapshots were extracted from each trajectory when the systems reached the geometrical stability, i.e. the RMSD *vs* time plot reached a plateau and the exponential tendency line of the peptide Caplha RMSF did not show any inclination (Figure S7). Twenty snapshots were regularly extracted to ensure the lowest standard error in the free energy estimation and the lowest calculation time. The time intervals for the extraction were chosen dividing by 20 the number of frames in which the systems showed the geometrical stability. MM-GBSA calculations were performed by *MM-PBSA.py* module of Amber12, keeping parameters in the default values. Nevertheless, this evaluation did not take into account the entropic contributions to the final binding free energy values, but only the enthalpy and desolvation free energy contributions. Thus, the obtained results should be considered as an attempt to estimate the binding energy values (ΔG*) of the lupin peptides under investigation^72^.

### Synthetic peptides

**T9** GQEQSHQDEGVIVR and **P5** LILPKHSDAD were synthesized by the company PRIMM (Milan, Italy). They were >95% pure by HPLC.

### *In vitro* PCSK9-LDLR binding assay of T9 and P5

Synthetic peptides, i.e. **T9** (32–320 μM) and **P5** (0.001–100 μM), were tested using the *in vitro* PCSK9-LDLR binding assay (CycLex Co., Nagano, Japan) following the manufacture instructions as reported above. The absorbance at 450 nm was measured using the Synergy H1 fluorescent plate reader (Biotek, Bad Friedrichshall, Germany). In particular, for the *in vitro* screening of the synthetic PCSK9-LDLR inhibitors, **T9** and **P5**, at different concentrations, were added to the appropriate amount of His-tagged PCSK9 in the wells that had been coated with recombinant LDLR-AB domain in a similar fashion as described above, followed by evaluation of inhibitory effect on PCSK9-LDLR interaction by measuring the amount of His-tagged PCSK9 on the wells which is correlated to the absorbance signals at 450 nm, which were measured using the Synergy H1 fluorescent plate reader (Biotek, Bad Friedrichshall, Germany).

### Cell line culture

The HepG2 cell line was bought from ATCC (HB-8065, ATCC from LGC Standards, Milan, Italy). HepG2 cells were cultured in DMEM high glucose with stable L-glutamine supplemented with 10% FBS, 100 U/ml penicillin, 100 μg/ml streptomycin (complete growth medium) and incubated at 37 °C under 5% CO_2_ atmosphere. HepG2 cells were used for no more than 20 passages after thawing, because the increase of the number of passages may change the cell characteristics and impair assay results.

### Fluorescent LDL uptake cell based assay

HepG2 cells (3 × 10^4^/well) were seeded in 96-well plates and kept in complete growth medium for 2 d before treatment. The third day, they were treated with **T9** (160 and 320 μM) or **P5** (10 μM), or vehicle (H_2_O) for 24 h. At the end of the treatment, the culture medium was replaced with 50 μl/well LDL-DyLight™ 550 working solution (Cayman Chemical Company, Ann Arbor, MI, US). The cells were additionally incubated for 2 h at 37 °C and then the culture medium was aspirated and replaced with PBS (100 μl/well). The degree of LDL uptake was measured using the Synergy H1 fluorescent plate reader from Biotek (excitation and emission wavelengths 540 and 570 nm, respectively).

### Statistical analysis of biological assays

Data are presented as mean ± standard error using GraphPad Prism 6 (GraphPad, La Jolla, CA, USA). Statistical analyses were carried out by one-way ANOVA followed by Dunnett’s test. P-values < 0.05 were considered to be significant.

### Quantification by mass spectrometry of peptide P5 in P-abs

The LC-M analysis was carried out in positive ionization mode on a SL series Ion Trap mass spectrometer equipped with a nESI source interfaced with a HPLC-Chip Cube source (Agilent Technologies, Palo Alto, CA, USA). The chromatographic separation was performed on a LC-chip containing a 40 nL enrichment column (Zorbax 300SB-C18, 5 μm pore size), a 43 mm × 75 μm analytical column packed (Zorbax 300SB-C18, 5 μm pore size). The sample (1 μL) was loaded onto the enrichment column at a flow rate 4 μL/min for 2 min using isocratic 100% C solvent phase (99% water, 1% ACN and 0.1% FA). After cleanup, the chip valve was switched to separation and trapped peptides were eluted into the mass spectrometer at the constant flow rate of 0.3 μL/min using H_2_O, 0.1% HCOOH (A) and ACN, 0.1% HCOOH (B) as elution solvent. The nano-pump gradient used was the following: 3% solvent B (0 min), 30% solvent B (0–4 min) and back to 3% in 1 min. A column re-equilibration time of 2 min was used after each analysis. The nano-ESI source operated under the following conditions: drying gas temperature 300 °C, flow rate 3 L/min (nitrogen), capillary voltage 1950 V, with endplate offset −500V. The LC-MS/MS analysis, under pseudo MRM condition, were carried out monitoring one diagnostic transition m/z 554.8 → m/z 770.0, selecting the most intense y-fragment ion, corresponding to y_7_. The QuantAnalysis data package was used to build calibration curves by integrating at each calibration level (CL) the area of the m/z 770.0, the main product ion of double charged m/z 554.8 precursor ion. The concentration was estimated using an external calibration curve built from standard solution of peptide. The concentration of analyte in the standard solution ranged from 5 to 50 ng/μL. The accuracy test calculated preparing one spiked sample at 8 ng/μl was higher than 110%. The repeatability of the method was obtained by injecting the sample and the calibration levels three times each. The amount of LILPKHSDAD in **P-abs** was 28.64 ± 0.43 ng/μl. The relative standard deviation (RSD %) value resulted 1.5%. The limit of detection (LOD) and the limit of quantification (LOQ) were calculated by applying the Eqs. S_LOD_ = S_RB_ + 3 σ_RB_ and S_LOQ_ = S_RB_ + 10 σ_RB_, respectively, following the directives of IUPAC and American Chemical Society’s Committee in Environmental Analytical Chemistry, where S_LOD_ is the signal at the limit of detection, S_LOQ_ is the limit of quantification, S_RB_ is the signal of blank control, and σ_RB_ is the standard deviation. The calculated values of LOQ and LOD, corresponding to 3.92 and 3.71 ng/μl, respectively, confirm the reliability of the quantification method.

## Additional Information

**How to cite this article**: Lammi, C. *et al*. Lupin Peptides Modulate the Protein-Protein Interaction of Pcsk9 with the Low Density Lipoprotein Receptor in Hepg2 Cells. *Sci. Rep.* **6**, 29931; doi: 10.1038/srep29931 (2016).

## Supplementary Material

Supplementary Information

## Figures and Tables

**Table 1:** Peptides identified in the basolateral compartment of Caco-2 cells grown in two compartment systems^36^.

Peptide	Sequence	Uniprot Code	Protein Source	Proteolytic Enzyme
**T1**	IILGNEDEQEYEEQR	Q6EBC1	β2-conglutin	Trypsin
**T2**	AVNELTFPGSAEDIER	Q6EBC1	β2-conglutin	Trypsin
**T3**	IVEFQSKPNTLILPK	Q6EBC1	β2-conglutin	Trypsin
**T4**	INEGALLLPHYNSK	Q6EBC1	β2-conglutin	Trypsin
**T5**	HSDADYVLVVLNGR	Q6EBC1	β2-conglutin	Trypsin
**T6**	SNEPIYSNK	Q6EBC1	β2-conglutin	Trypsin
**T9**	GQEQSHQDEGVIVR	Q53HY0	β1-conglutin	Trypsin
**T10**	LLGFGINAYENQR	Q53HY0	β1-conglutin	Trypsin
**T13**	LNALEPDNTVQSEAGTIETWNPK	Q53I54	α-conglutin	Trypsin
**T16**	QEEQLLEQELENLPR	Q333K7	δ-conglutin	Trypsin
**P1**	YEITPDRNPQVQDL	Q6EBC1	β2-conglutin	Pepsin
**P3**	YDFYPSSTKDQQS	Q6EBC1	β2-conglutin	Pepsin
**P5**	LILPKHSDAD	Q6EBC1	β2-conglutin	Pepsin
**P6**	LRIPAGSTSY	Q6EBC1	β2-conglutin	Pepsin
**P7**	LTFPGSAED	Q6EBC1	β2-conglutin	Pepsin

**Table 2:** Results of MM-GBSA calculations on PCSK9/peptide complexes.

Peptide	Sequence	ΔG_MM-GBSA_ ± SE ΔG = G_Complex_ − (G_PCSK9_ + G_Peptide_) [kcal/mol]
**T9**	GQEQSHQDEGVIVR	−46.1 ± 1.0
**T1**	IILGNEDEQEYEEQR	−42.0 ± 0.9
**P5**	LILPKHSDAD	−39.1 ± 0.8
**P3**	YDFYPSSTKDQQS	−37.4 ± 1.2
**T10**	LLGFGINAYENQR	−31.1 ± 0.8
**T4**	INEGALLLPHYNSK	−25.9 ± 1.2
**T5**	HSDADYVLVVLNGR	−20.0 ± 1.4
**T3**	IVEFQSKPNTLILPK	−19.7 ± 0.8
**P6**	LRIPAGSTSY	−16.3 ± 1.1
**P1**	YEITPDRNPQVQDL	−15.6 ± 0.9
**T13**	LNALEPDNTVQSEAGTIETWNPK	−10.9 ± 2.7
**T2**	AVNELTFPGSAEDIER	−8.6 ± 0.5
**T6**	SNEPIYSNK	−8.1 ± 0.5
**P7**	LTFPGSAED	−8.2 ± 0.7
**T16**	QEEQLLEQELENLPR	−4.7 ± 1.4
