# Supplementary material for: Lupin Peptides Modulate the Protein-Protein Interaction of PCSK9 with the Low Density Lipoprotein Receptor in HepG2 Cells
Source: Sci Rep. 2016 Jul 18;6:29931. doi: 10.1038/srep29931 (PMC4947907; doi:10.1038/srep29931)
Supplement: Supplementary Information [file srep29931-s1.pdf]

Supporting Information

LUPIN PEPTIDES MODULATE THE PROTEIN-PROTEIN INTERACTION OF PCSK9 WITH THE LOW DENSITY LIPOPROTEIN RECEPTOR IN HEPG2 CELLS

Carmen Lammi, Chiara Zanoni, Gilda Aiello, Anna Arnoldi, Giovanni Grazioso

Department of Pharmaceutical Sciences, University of Milan, Via L. Mangiagalli 25, 20133 Milan, Italy

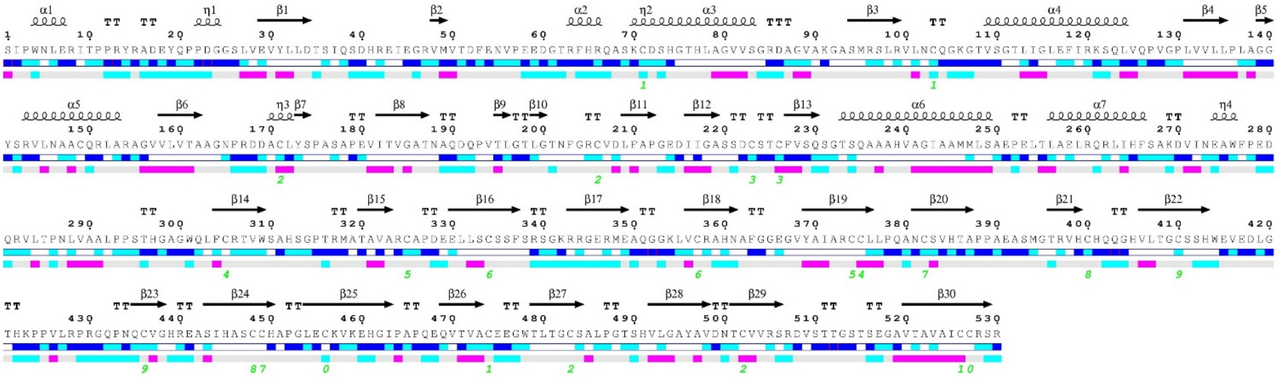

Figure S1. Sequence and secondary structure information of PCSK9 model. Here, green numbers indicate the SS bridges position in the sequence.

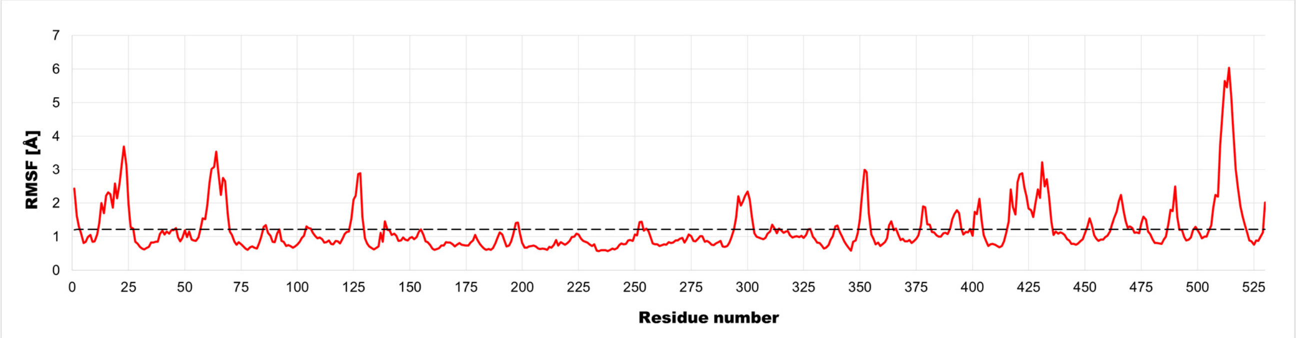

Figure S2. RMSF of PCSK9 Cα atoms *vs.* the mean coordinates. Black dotted line highlight the mean RMSF value (1.21 Å).

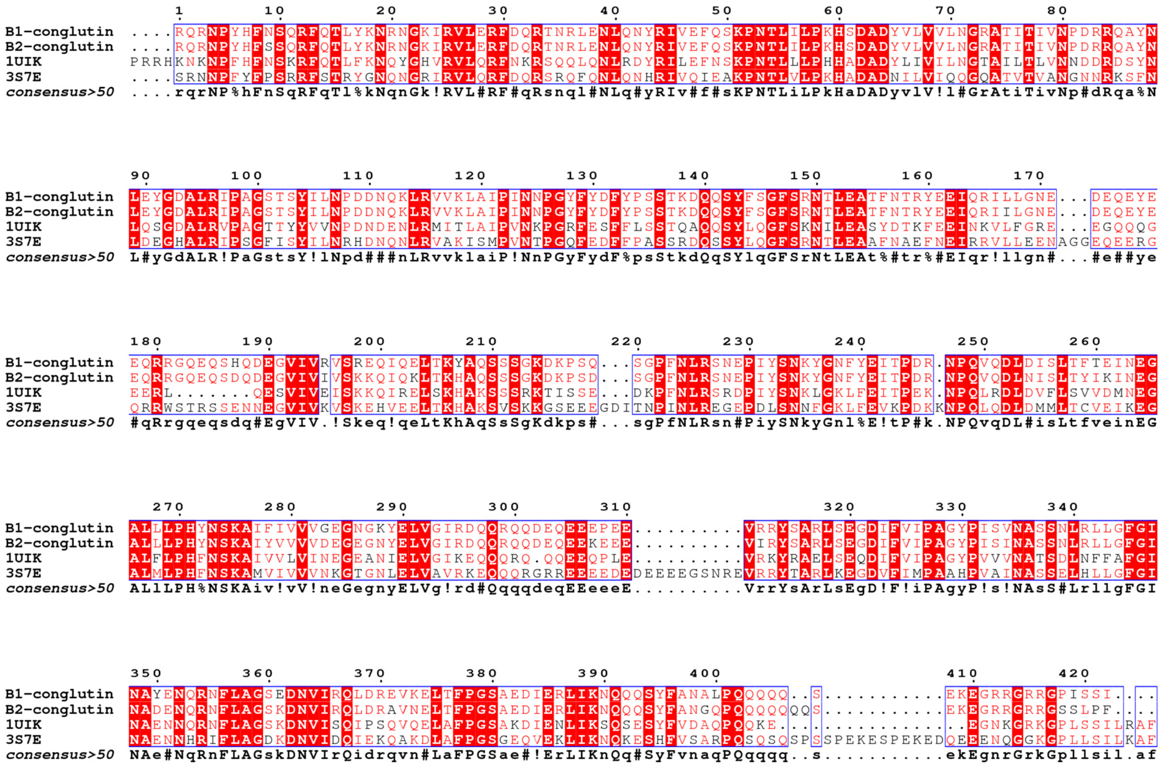

Figure S3. Sequence alignment of β1-, β2-conglutins and the sequences of soybean beta-conglycinin (PDB code 1UIK) and AraH1 peanut vicilin-like protein (PDB code 3S7E). Figure was acquire by ESPript server (http://endscript.ibcp.fr/ESPript/ESPript/index.php).

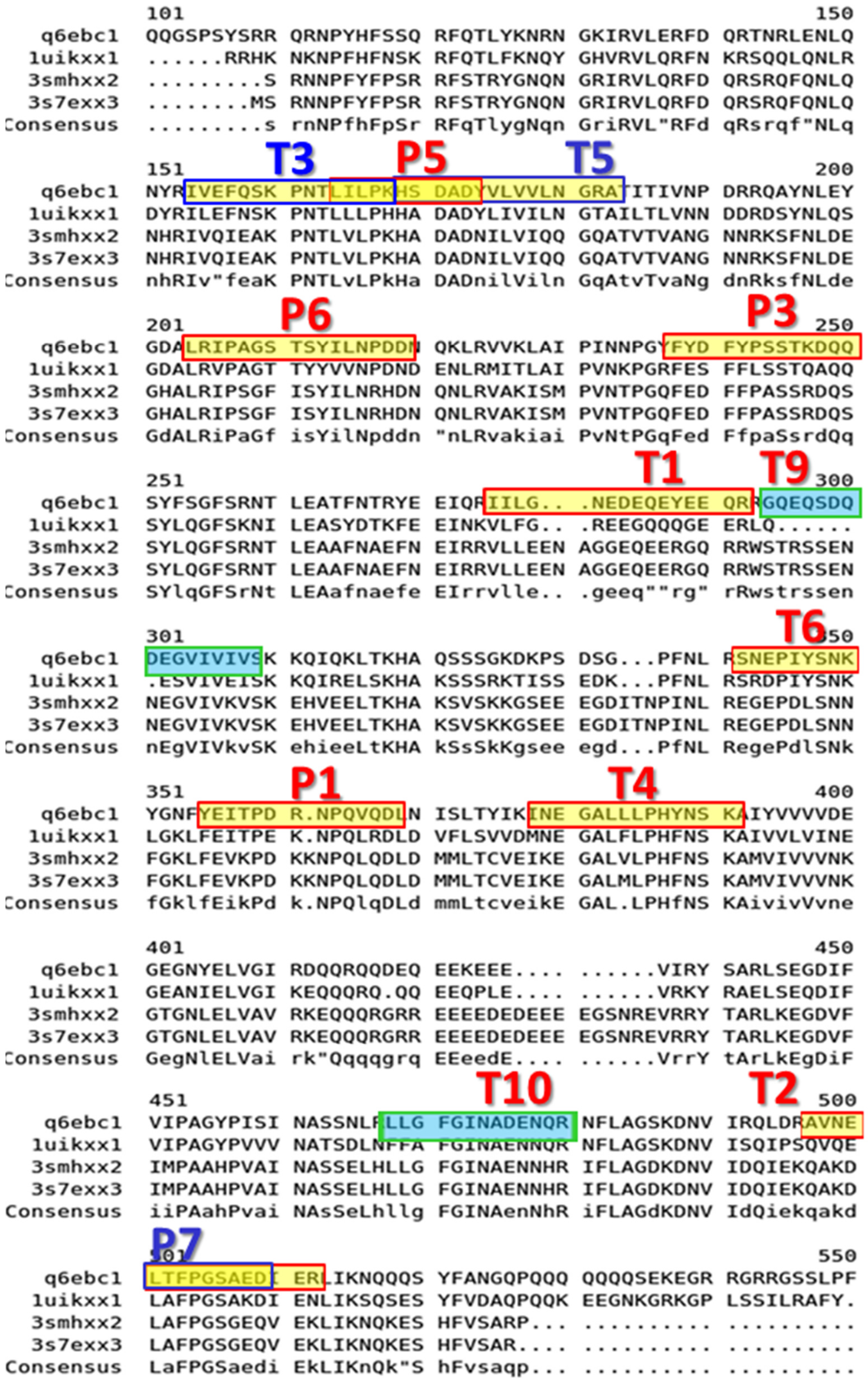

Figure S4. Sequence alignment of β2-conglutins (UniProt code Q6EBC1) and the sequences of soybean beta-conglycinin (PDB code 1UIK) and peanut AraH1 vicilin-like protein (PDB code 3S7E, and 3SMH). Numbers represent the small-peptides sequences further extracted to create the suitable models.

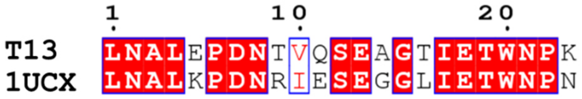

Figure S5. Sequence alignment between peptide **T13**, deriving from α-conglutin, and the soybean proglycinin (PDB codes 1UCX).

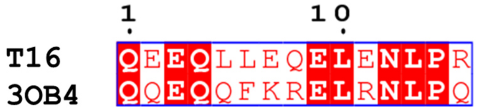

Figure S6. Sequence alignment of peptide **T16**, deriving from δ-conglutin, and peanuts AraH2 protein (PDB code 3OB4)

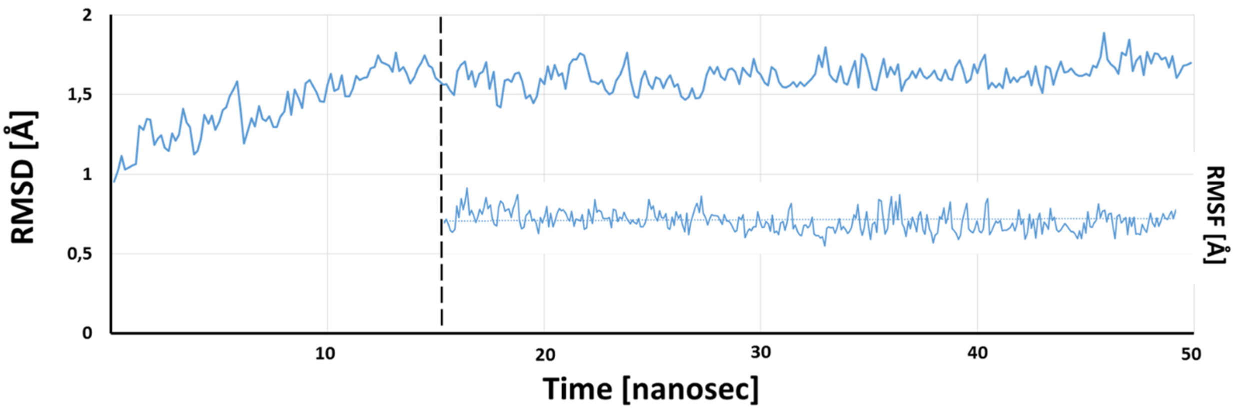

A

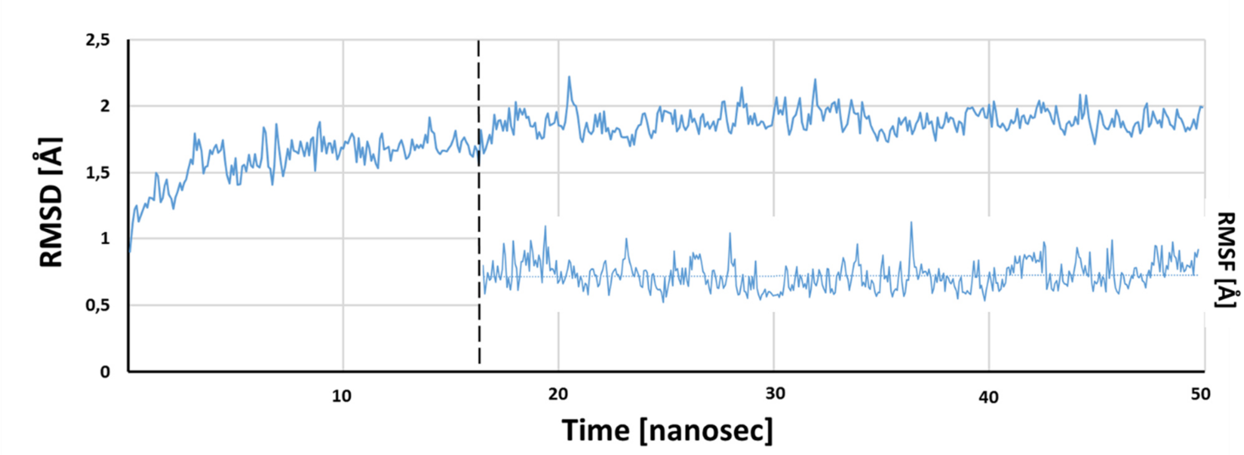

B

Figure S7. RMSD (Å) over simulation time of the **P5** (panel A) and **T9** (panel B) Cα atoms aligned on the equilibrated structures. The RMSF of the frames considered for the MM-GBSA calculations are also shown in the plot under the previous one. The exponential tendency lines are showed as dotted line.
